# Supplementary material for: A PCR Test Using the Mini-PCR Platform and Simplified Product Detection Methods Is Highly Sensitive and Specific to Detect Fasciola hepatica DNA Mixed in Human Stool, Snail Tissue, and Water DNA Specimens
Source: Pathogens. 2024 May 23;13(6):440. doi: 10.3390/pathogens13060440 (PMC11206539; doi:10.3390/pathogens13060440)
Supplement: Supplementary file 1 [file pathogens-13-00440-s001.zip › pathogens-3014642-supplementary_fIGURE 1.pdf]

Serial dilutions LOD Fh + H2O

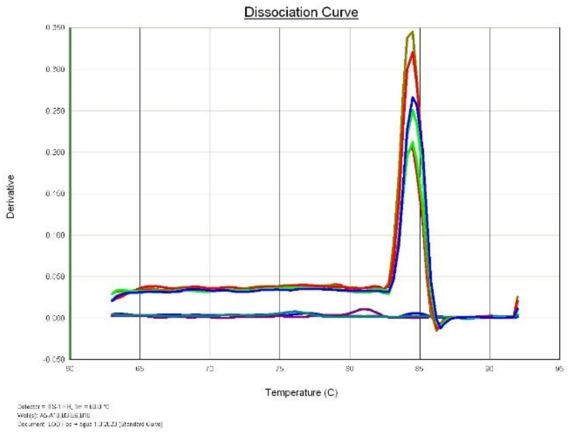

Serial dilutions LOD Fh + stools

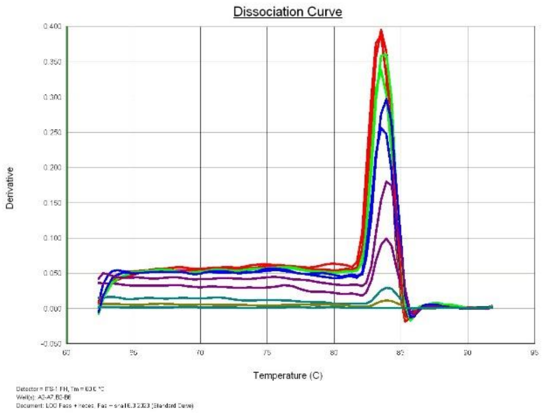

Serial dilutions LOD Fh + Snail

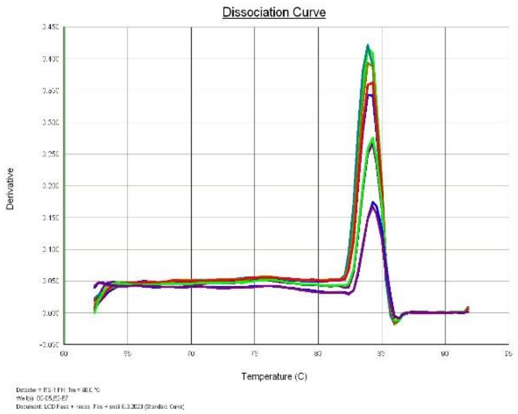

Supplemental data Figure S1. "Dissociation curves of real time PCR experiments to detect *Fasciola* DNA". Dissociation curves a specific peak at 84 C°, no dimer amplification or dissociation curves were observed in negative samples. The real time PCR amplification was conducted using specific primers for *Fasciola* 18s rDNA gene previously characterized [13]. Abbreviations: Fh = *Fasciola hepatica*
