# Supplementary material for: A PCR Test Using the Mini-PCR Platform and Simplified Product Detection Methods Is Highly Sensitive and Specific to Detect Fasciola hepatica DNA Mixed in Human Stool, Snail Tissue, and Water DNA Specimens
Source: Pathogens. 2024 May 23;13(6):440. doi: 10.3390/pathogens13060440 (PMC11206539; doi:10.3390/pathogens13060440)
Supplement: Supplementary file 1 [file pathogens-13-00440-s001.zip › pathogens-3014642-supplementary_TABLE_1.pdf]

Supplementary Table S1. Relative units Intensity (RUI) in pictures taken from P51 mini viewer.

Sample	+	+	+	+	-	-	-	-
RUI Rep1	177	171	169	170	130	123	128	135
RUI Rep2	166	184	178	162	125	126	130	127
RUI Rep3	159	164	166	164	133	133	129	130
Avg	167.3333	173	171	165.3333	129.3333	127.3333	129	130.6667
sd	8.115828	9.077445	5.585696	3.723797	3.614784	4.589844	0.894427	3.614784

RUI FAS miniPCR (Fig 2C)

RUI Water (Fig. 4C)

Sample	10 pg	1 pg	100 fg	10 fg	1 fg	no template
Rep1	133	153	126	135	97	61
Rep2	142	121	129	117	96	65
Rep 3	151	117	127	127	121	62
Av	142	130.3333	127.3333	126.3333	104.6667	62.66667
Sd	9	19.73153	1.527525	9.0185	14.15392	2.081666

RUI stool (Fig 5C)

Sample DNA	1 ng	100 pg	10 pg	1 pg	100 fg	10 fg	1 fg	NC
RUI Rep1	131	137	130	137	133	78	72	71
RUI Rep2	140	131	148	131	130	114	107	77
RUI Rep3	129	126	126	132	126	70	98	65
Av	133.333	131.333	134.666	133.3	129.66	87.3333	92.3333	71
sd	5.85946	5.5075	11.718	3.21455	3.511885	23.43786	18.17507	6

RUI Snail tissue (Fig 6C)

RUI Rep1	107	135	116	114	102	108	77	55
RUI Rep2	119	124	123	113	115	99	72	76
RUI Rep3	111	111	100	120	105	96	88	68
Avg	112.3333	123.3333	113	115.6667	107.3333	101	79	66.33333

Sd	6.1101	12.013	11.7898	3.785939	6.806859	6.244998	8.185353	10.59874

Specificity (Fig 7)

Sample	Fh	Hn	Tt	Ac	Al	Pc	Ts	Dp	Em	NT
Rep 1	177	148	148	133	129	158	150	156	146	153
Rep 2	174	138	140	132	140	141	143	152	126	133
Rep 3	216	149	130	121	127	133	143	142	150	159
Avg.	189	145	139.3	128.67	132	144	145.33	150	140.667	148.3333
Std	23.4	6.08	9.01	6.65	7	12.76	4.04	7.21	12.8	13.612
